# The crowding out effect of out-of-pocket medication expenses of two major non-communicable diseases in Pakistan

**DOI:** 10.1093/inthealth/ihz075

**Published:** 2018-10-14

**Authors:** Biplab K Datta, Muhammad J Husain, Sohani Fatehin

**Affiliations:** Global Noncommunicable Diseases Branch, Division of Global Health Protection, Center for Global Health, Centers for Disease Control and Prevention, 1600 Clifton Road NE, MS V18-3, Atlanta, GA , USA; Global Noncommunicable Diseases Branch, Division of Global Health Protection, Center for Global Health, Centers for Disease Control and Prevention, 1600 Clifton Road NE, MS V18-3, Atlanta, GA , USA; Department of Economics, Dickinson College, 28 N College Street, Carlisle, PA 17013, USA

**Keywords:** diabetes, drug cost, household consumption, hypertension, non-communicable diseases, out-of-pocket expenditure

## Abstract

**Background:**

Elevated blood pressure (i.e. hypertension) and diabetes (BPD) are the two major noncommunicable diseases that expose households to high out-of-pocket treatment costs in low- and middle-income countries. Medication is the biggest share of BPD treatment expenses, and households with someone suffering from BPD may need to adjust consumption of other commodities to pay for essential BPD medicines. We assess how BPD medication expenditures are associated with crowding out of other household commodities in Pakistan.

**Methods:**

We analyze self-reported household consumption data from the nationally representative Pakistan Household Income and Expenditure Survey 2015–16. We estimate conditional Engel curves under the Quadratic Almost Ideal Demand System framework to examine the differences in average consumption shares between BPD medication-consuming and not-consuming households.

**Results:**

We find that BPD medication expenditures are associated with crowding out of food and crowding in of other medical expenditures for all households, but the magnitudes of crowding out and crowding in are larger for the poorer households. BPD medication spending is also associated with crowding out of education and personal care for middle-class and wealthier households.

**Conclusions:**

Our results indicate that allocations for essential commodities, like food and education, are lower for BPD medication–consuming households and inform policies for preventive health promotions and affordable treatment for hypertension and diabetes.

## Introduction

The increasing health and economic burdens of deaths and disabilities from noncommunicable diseases (NCDs) are emerging as major concerns worldwide, particularly for low- and middle-income countries (LMICs).^1–4^ NCDs are responsible for 41 million deaths annually, equivalent to 71% of all deaths worldwide; 15 million of those deaths are premature, i.e. between the ages of 30 and 69 y.^5^^,^^6^ Cardiovascular diseases (CVDs) account for the majority of NCD deaths (17.9 million people annually), followed by cancers (9.0 million), respiratory diseases (3.9 million) and diabetes (1.6 million).^5^ Hypertension (or elevated blood pressure [BP]), the key risk factor for CVD, accounts for about half of the CVD deaths, affecting 1 billion people worldwide and killing an estimated 9.4 million people every year.^7^

CVD alone is estimated to cost US$20 trillion in healthcare costs and productivity losses globally over the 20 y period from 2011 to 2030, a major portion of which can be attributed to hypertension-associated complications.^8^ In contrast, diabetes cost the global economy nearly US$500 billion in 2010, which is expected to increase to an estimated US$745 billion in 2030, with developing countries increasingly taking on a much greater share of the outlays. NCDs thus pose major challenges for sustainable development for the twenty-first century.^1^^,^^2^

At the household level, NCDs cause enormous financial burden due to direct medical costs (e.g. medical consultations, medicines, diagnostic tests, hospital stays), nonmedical costs (e.g. transportation, adjustments to household amenities) and indirect costs from lost income and productivity for the patients and caregivers.^9^ Families incur high out-of-pocket (OOP) expenditures, often long term in the case of NCD complications, pushing millions of people into poverty.^7^ This could impact a household’s resource allocation, with adverse consequences for food and nutrition and human capital development (e.g. education spending).^4^^,^^10^

Previous studies have mostly examined the link between NCDs and the odds of catastrophic health expenditures and impoverishment in the LMICs.^11^^,^^12^ Other studies have examined how overall OOP health expenditures or healthcare utilization impact household consumption.^13^^,^^14^ Little is known about the impact of OOP NCD treatment expenditures on household resource allocation. In this article we focus on two major NCDs, hypertension and diabetes, and investigate how medication costs for treating these diseases are associated with household consumption patterns in the LMIC context. More specifically, using data from a nationally representative self-reported household survey, this article explores the role of OOP medication expenses for BP and diabetes in household resource allocation in Pakistan. To our knowledge, this is the first article examining this relationship in the LMIC context.

Pakistan is a South Asian lower middle-income country with a large population of 195 million and a per capita income of US$1531 in 2015–16. According to the Pakistan National Health Survey, 33% of Pakistani people >45 y of age have elevated BP.^15^ Pakistan also ranks sixth in terms of the number of people with diabetes worldwide.^16^ The prevalence of diabetes in Pakistan is 9.8%.^17^ Diabetes and hypertension together constitute 7.6% of the total health expenditures (THEs) of Pakistan in 2013–14. Private households bear the major burden of healthcare costs, as 60% of the THEs are financed by households’ OOP expenditures. And medicines, with a >52% share of OOP expenditures, is by far the largest component in households’ OOP spending.^18^ A recent study showed that the average medical expenditures are significantly higher for households spending on hypertension and diabetes medication in Pakistan, and the medication costs for managing these two major NCDs are strongly associated with household catastrophic health expenditures.^19^ The high prevalence of hypertension and diabetes and the OOP treatment costs of these diseases are therefore a major public health concern in Pakistan. These research findings could be relevant for policymakers in evaluating poverty alleviation and human development strategies through low-cost healthcare, preventive health promotions and affordable treatment for hypertension and diabetes.

## Methods

### Data

This study uses data from the Household Integrated Economic Survey (HIES) 2015–16 of Pakistan. The HIES 2015–16 is a nationally representative survey of 16155 urban and 8083 rural households that collects detailed self-reported information on household income and consumption expenditures.^20^ The HIES collects household-level consumption expenditures of 454 nondurable goods and services. Households report how much they spend fortnightly, monthly or annually, depending on the type of the commodity. We calculate the household’s monthly consumption expenditure by aggregating paid consumptions of nondurable commodities in 11 broad categories: food, tobacco, clothing, housing and household effects, education, fuel and electricity, personal care, transport and communication, recreation, miscellaneous and medical. Following the ‘balance sheet for income and expenditure’ in the HIES questionnaire, the calculation of total expenditures does not include consumption values that are unpaid (e.g. wages and salaries received in kind, own produced, receipt from assistance, gift or other sources). Among the items under the medical category, households were asked about yearly consumption expenditures on tablets for BP and tablets and insulin for blood sugar or diabetes. We define households as a BP and/or diabetes (BPD) medication–consuming household if they report positive spending on these items. Monthly BPD medication expenditures are subtracted from the medical category to create another category called ‘other medical’.

### Conceptual background

We do not study the impact of hypertension and diabetes morbidity on household resource allocation; rather, we investigate the contemporaneous association between BPD medication expenditures and household resource allocation. BPD morbidity may be associated with other comorbidities (e.g. kidney diseases), and BPD morbidity and related comorbidities could affect a household’s resource allocation via loss of income or productivity. BPD morbidity and related comorbidities could also have direct impacts on household resource allocation because of adjustments in dietary behaviors and lifestyle practices. Certain household consumptions (e.g. food, alcohol, tobacco), on the other hand, could influence BPD morbidity and related comorbidities as well. These channels, which are subject to intertemporal analysis, are not studied in this article.

We investigate, if BPD is treated via medication, how medication expenditures and household resource allocations are associated at a point of time. We assume that if BPD is treated, then households first allocate a certain amount of income for BPD medication consumption as required and then reallocate the rest of the income for consumption of other commodities. BPD diagnosis/treatment, however, depends on a household’s socioeconomic status, which along with household demography, directly affects resource allocation patterns. We analyze the relationship between BPD medication expenditures and household resource allocation by conditioning on household demography and socioeconomic status. Our study is specific to how BPD treatment expenditures (in terms of medication costs) and household consumption are related in cross-section data. We estimate the contemporaneous relationship between BPD medication expenditures and household resource allocation and do not analyze any intertemporal aspects of this association.

Our analysis entails the conditional demand function framework formally developed by Pollak.^21^ There is a ‘preallocated’ good in the household’s consumption basket, which in our case is the BPD medication. Households consuming BPD medication first allocate the necessary amount of income to purchase the required quantity of BPD medication and then spend the remaining income on other commodities. In this setting, the utility maximization problem of a representative household is

\begin{eqnarray*}
\operatorname{Max}\ \mathrm{U}\left({\mathrm{x}}_1,{\mathrm{x}}_2,\dots, {\mathrm{x}}_{\mathrm{n}-1},{\mathrm{x}}_{\mathrm{n}}\right)
\end{eqnarray*}

 

(1) \begin{eqnarray*}
{\textrm{s.t.}}\ \sum_{j=1}^{n-1}{p}_j{x}_j=Y
\end{eqnarray*}

 

\begin{eqnarray*}
{x}_n={x}_n^{\ast },
\end{eqnarray*}

where *p_j_* is the price of the *j*th good, x^*^_n_ is the quantity of BPD medication and *Y* is the total expenditure on goods that are not preallocated (i.e. x_1_, x_2_, …, x_n−1_). For households consuming BPD medication, *Y = I*−*p_n_x_n_*, where *I* is the total household expenditures. For households not consuming BPD medication, x^*^_n_ = 0 and *Y* = *I*. The conditional Marshallian demand for the *j*th good, which depends on the quantity of the preallocated good, prices of all goods except the preallocated good and total expenditures net expenditure on the preallocated good, can be expressed as

(2) \begin{eqnarray*}
{x}_j={x}^j\left({p}_1,{p}_2,\dots, {p}_{n-1},Y,{x}_n^{\ast}\right).
\end{eqnarray*}

Weak separability of other commodities from BPD medication implies an income effect of BPD medication expenditures on the consumption of other commodities.^22^ We examine how consumption differs between BPD medication-consuming and not-consuming households due to this income effect. Due to the cross-sectional nature of our data, we do not observe price changes and assume that all households (within a geographic area) face similar prices for certain commodities. Therefore, instead of estimating a demand system, we estimate Engel curves for broad commodity groups (e.g. food, education) to analyze the association between BPD medication expenditures and household consumption. Previous studies use a similar framework to analyze the impact of OOP health expenditures,^13^ tobacco expenditures,^23^ etc. on household resource allocation.

### Differences in consumption share

We first compare differences in average expenditure shares across broad consumption categories between households consuming and not consuming BPD medication. While calculating expenditure shares, BPD medication spending is subtracted from total expenditures, so that we observe how households reallocate resources across consumption categories after spending on BPD medication. Since households’ spending patterns vary across income groups (e.g. wealthier households spend relatively less on food than poor households), we observe the differences across five consumption (per capita) quintiles. Consumption quintiles refer to the household’s socioeconomic status and are estimated based on the household’s per capita total consumption of nondurable commodities. However, these differences are not adjusted for household demographic and other socioeconomic characteristics. To account for variations in household characteristics, we estimate conditional Engel curves derived from the Quadratic Almost Ideal Demand System (QAIDS) framework, proposed by Banks et al.^24^ Under this framework, commodities can be luxury or necessity, depending on the household’s income level. The QAIDS framework allows us to examine any differential impacts of BPD medication spending on household resource allocation for households of a different economic status. Using the seemingly unrelated regression (SUR) model, we estimate the following system of equations:

(3) \begin{eqnarray*}
{w}_{ij}&=&{\alpha}_0+{\alpha}_1 BP{D}_i+\left({\alpha}_2+{\alpha}_3 BP{D}_i\right)\ln {Y}_i\\
&&+\left({\alpha}_4+{\alpha}_5 BP{D}_i\right){\left(\ln {Y}_i\right)}^2+{\boldsymbol{X}}_{\boldsymbol{i}}{\boldsymbol{\alpha}}_{\mathbf{6}}+\boldsymbol{P}{\boldsymbol{R}}_{\boldsymbol{i}}\boldsymbol{\lambda} +{\varepsilon}_{ij},
\end{eqnarray*}

where *w_ij_* is the consumption expenditure share of the *j*th consumption category of household *i*. Since consumption shares add up to 100%, the miscellaneous consumption category is omitted from the system of equations to meet the summation restriction. *BPD_i_* is a dummy that takes the value 1 if household *i* reports positive spending on BPD medication and 0 otherwise. *Y_i_* is household *i*’s consumption expenditure net BPD medication. Since our outcome variables are expenditure shares, which include *Y_i_* in the denominator, controlling for *Y_i_* may raise endogeneity concerns. To overcome potential endogeneity issues, following Banks et al.^24^ and Pal,^13^ the log of household consumption expenditures, ln *Y_i_*, and the log of household consumption expenditures squared, (ln *Y_i_*)^2^, are respectively instrumented by the log of household income, ln *M_i_*, and the log of household income squared, (ln *M_i_*)^2^. The interaction terms of ln *Y_i_* and (ln *Y_i_*)^2^ are also instrumented by the products of *BPD_i_* and ln *M_i_* and *BPD_i_* and (ln *M_i_*)^2^, respectively.

***X***_***i***_ in equation 3 is a vector of household-level characteristics, including the share of children <5 y of age in the household, the share of elderly (age ≥65 y) in the household, whether the household has school-age children (ages 6–15 y), whether the household has reproductive age females (ages 15–49 y), household size, household head’s education, whether the household has a gas connection, the household’s source of drinking water, the household’s type of toilet, whether the household has a computer/laptop/tablet, whether the household has internet access, whether the household receives a government cash transfer (Benazir Income Support Programme [BISP]), whether the household receives remittances from abroad, the household’s type of agricultural production, whether the household owns livestock, whether the household owns poultry and whether the household borrowed to finance consumption expenditures during the survey period. ***PR***_***i***_ controls for province/region fixed effects that account for price differences and other province-/region-specific consumption preferences. Lastly, *ε_ij_* is the idiosyncratic error term. There are four provinces (Punjab, Sindh, Balochistan and Khyber Pakhtunkhwa) and three regions (large cities, other urban and rural) in the data. We assume that households within the same province/region face similar prices for respective commodities.

The adjusted difference in the consumption expenditure share between BPD medication–consuming and not-consuming households is given by equation 4:

(4) \begin{eqnarray*}
E\left[{w}_{ij}| BP{D}_i=1\right]-E\left[{w}_{ij}| BP{D}_i=0\right]={\alpha}_1+\ {\alpha}_3\times \ln {Y}_i\ +\ {\alpha}_5\times {\left(\ln {Y}_i\right)}^2.
\end{eqnarray*}

Under the QAIDS framework, the differences not only depend on *α_1_*, the coefficient of *BPD_i_* from equation 3, but also on *α_3_*, *α_5_* and the level of household expenditure, *Y_i_*. Therefore the sign and magnitude of adjusted differences could vary across the household’s economic status. We calculate and report adjusted differences for the consumption categories for which we get statistically significant estimates (i.e. estimated coefficients are statistically different from 0) of α_1_, α_3_ and α_5_ at the 5th, 10th, 20th, 30th, 40th, 50th, 60th, 70th, 80th, 90th and 95th percentiles of monthly household per capita expenditures.

### Crowding out

Next we analyze the crowding out effect of BPD medication spending. We define crowding out (or crowding in) as the marginal effect of BPD medication spending on the expenditure share of a particular consumption category. Crowding out occurs if spending on BPD medication is associated with a reduction in the expenditure share of a consumption category. The crowding out effect is estimated by the system of equations as follows:

(5) \begin{eqnarray*}
{w}_{j} & = & {\beta}_0+{\beta}_1 BP{DMX}_i+\left({\beta}_2+{\beta}_3 BP{DMX}_i\right)\ln {Y}_i+\\
&& \left({\beta}_4+{\beta}_5 BP{DMX}_i\right){\left(\ln {Y}_i\right)}^{2}+{\boldsymbol{X}}_{\boldsymbol{i}}{\boldsymbol{\beta}}_{\mathbf{6}}+\boldsymbol{P}{\boldsymbol{R}}_{\boldsymbol{i}}{\boldsymbol{\lambda}} +{\mu}_{ij},
\end{eqnarray*}

where *BPDMX_i_* is BPD medication spending of household *i*. For households that do not consume BPD medication, *BPDMX_i_* is 0. The estimation method and control variables in equation 5 are the same as those in equation 3. Crowding out is formally defined as a decrease in the share (percentage point) of consumption category *j* due to a 1 Pakistani rupee (Rs) increase in BPD medication expenditures:

(6) \begin{eqnarray*}\frac{\delta {w}_{ij}}{\delta BP{DMX}_i}={\beta}_1+{\beta}_3\times \ln {Y}_i+{\beta}_5\times {\left(\ln {Y}_i\right)}^2.
\end{eqnarray*}

Therefore BPD medication expenditures crowd out consumption of category *j* if $\delta {w}_{ij}/\delta {BP{DMX}_{i}}$<0, and conversely crowd in consumption of category *j* if $\delta {w}_{ij}/\delta BP{DMX}_i$>0. If $\delta {w}_{ij}/\delta BP{DMX}_i$ = 0, then BPD medication spending has no direct impact on household resource reallocation.

Similar to adjusted differences, the crowding out effect under the QAIDS framework depends on coefficient estimates of β_1_, β_3_ and β_5_ and household expenditures *Y_i_*, and is calculated using equation 6. For statistically significant estimates of β_1_, β_3_ and β_5_ of consumption category *j*, we calculate and report crowding out or crowding in effects at the 5th, 10th, 20th, 30th, 40th, 50th, 60th, 70th, 80th, 90th and 95th percentiles of monthly household per capita expenditures.

## Results

On average, 25.4% of the households in Pakistan report positive spending on BPD medication. The proportion of BPD medication–consuming households is higher in urban areas (28.5%) than in rural areas (23.6%). BPD medication consumption incidence is higher in wealthier households, as the proportion gradually increases from 15.6% in the first quintile to 33.7% in the fifth quintile of total household consumption. A typical BPD medication spending household spends Rs438 (Rs1≈US$0.01), or 1.45% of the monthly household expenditures, on BPD medication. Average spending is also higher in the upper consumption quintiles (Rs668 in the top quintile compared with Rs245 in the bottom quintile).

Table 1 reports the average consumption share by consumption quintiles for households that do not consume BPD medication. Food has by far the largest share in the consumption pie across all consumption quintiles. However, the share of food is relatively higher in the lower quintiles and lower in the upper quintiles. In contrast, shares of housing, education, fuel and electricity and transport and communication are relatively higher in the upper quintiles and lower in the lower quintiles.

**Table 1:** Average consumption shares for households not consuming BPD medication, by consumption quintiles

Shares	Quintile 1	Quintile 2	Quintile 3	Quintile 4	Quintile 5
Food	59.17 (58.47 to 59.86)	55.71 (55.18 to 56.24)	53.06 (52.47 to 53.64)	50.26 (49.59 to 50.93)	43.48 (42.76 to 44.19)
Tobacco	1.89 (1.73 to 2.04)	1.86 (1.72 to 2.00)	1.60 (1.48 to 1.72)	1.57 (1.42 to 1.73)	1.13 (1.03 to 1.24)
Clothing	11.33 (10.96 to 11.71)	10.99 (10.70 to 11.28)	10.83 (10.57 to 11.09)	10.43 (10.17 to 10.69)	9.42 (9.20 to 9.64)
Housing	1.14 (0.97 to 1.30)	1.80 (1.59 to 2.00)	2.44 (2.20 to 2.67)	3.25 (2.90 to 3.61)	4.67 (4.21 to 5.13)
Education	1.85 (1.69 to 2.02)	2.55 (2.36 to 2.75)	3.44 (3.20 to 3.68)	4.40 (4.11 to 4.69)	6.91 (6.51 to 7.32)
Fuel	5.71 (5.37 to 6.05)	6.59 (6.25 to 6.92)	7.13 (6.85 to 7.41)	7.70 (7.37 to 8.03)	8.24 (7.91 to 8.57)
Personal care	6.88 (6.73 to 7.02)	6.69 (6.56 to 6.83)	6.47 (6.34 to 6.59)	6.28 (6.17 to 6.39)	5.78 (5.65 to 5.90)
Transport	4.48 (4.27 to 4.70)	5.59 (5.36 to 5.81)	6.35 (6.10 to 6.60)	7.16 (6.90 to 7.42)	9.08 (8.79 to 9.37)
Recreation	0.28 (0.22 to 0.35)	0.47 (0.38 to 0.57)	0.56 (0.48 to 0.64)	0.55 (0.49 to 0.60)	0.86 (0.74 to 0.98)
Miscellaneous	4.18 (3.95 to 4.42)	4.73 (4.50 to 4.97)	5.21 (4.96 to 5.46)	5.29 (5.03 to 5.55)	7.76 (7.30 to 8.22)
Other medical	3.09 (2.80 to 3.38)	3.02 (2.82 to 3.21)	2.93 (2.66 to 3.19)	3.11 (2.84 to 3.38)	2.66 (2.28 to 3.04)

Values presented as % (95% CI). Values in each column add up to 100. ‘Other medical’ is the total medical expenditures less BPD medication expenses. For households not consuming BPD medication, other medical expenditures is the same as the total medical expenditures. Quintile 1 refers to households in consumption quintile 1.

The unadjusted differences in consumption share between BPD medication–consuming and not-consuming households are reported in Table 2. In general, BPD medication–consuming households on average allocate less for food and personal care and more for clothing and transport and communication than households not consuming BPD medication across all consumption quintiles. Differences for housing and fuel shares are not statistically significant for any quintiles. At the two lower quintiles, BPD medication–consuming households allocate more for education; however, the differences are negative, although not statistically significant, in the three upper quintiles. Differences in the other medical share (i.e. total medical expenditures less BPD medication expenses) are positive but statistically significant for the second and fifth quintiles only.

**Table 2:** Unadjusted differences in average consumption shares between BPD-consuming and not-consuming households by consumption quintiles

Shares	Quintile 1	Quintile 2	Quintile 3	Quintile 4	Quintile 5
Food	−3.43^***^ (−5.00 to −1.85)	−2.70^***^ (−3.81 to −1.60)	−0.99^*^ (−2.06 to 0.07)	−1.35^**^ (−2.41 to −0.29)	−2.55^***^ (−3.47 to −1.63)
Tobacco	0.02 (−0.32 to 0.36)	−0.30^**^ (−0.60 to −0.01)	−0.24^**^ (−0.46 to −0.02)	−0.29^**^ (−0.52 to −0.05)	−0.11 (−0.26 to 0.05)
Clothing	1.29^***^ (0.52 to 2.06)	1.12^***^ (0.60 to 1.63)	0.41^*^ (−0.03 to 0.85)	0.42^**^ (0.02 to 0.82)	0.36^**^ (0.02 to 0.69)
Housing	−0.09 (−0.38 to 0.20)	0.19 (−0.21 to 0.59)	−0.12 (−0.53 to 0.29)	−0.32 (−0.78 to 0.15)	−0.40 (−1.02 to 0.21)
Education	0.37^**^ (0.09 to 0.65)	0.35^**^ (−0.07 to 0.77)	−0.27 (−0.71 to 0.17)	−0.14 (−0.59 to 0.30)	−0.10 (−0.69 to 0.48)
Fuel	−0.14 (−0.74 to 0.45)	0.02 (−0.54 to 0.58)	0.18 (−0.31 to 0.67)	−0.18 (−0.62 to 0.25)	−0.11 (−0.53 to 0.31)
Personal care	−0.45^***^ (−0.73 to −0.17)	−0.46^***^ (−0.69 to −0.23)	−0.23^**^ (−0.46 to 0.00)	−0.28^**^ (−0.52 to −0.04)	−0.27^***^ (−0.45 to −0.09)
Transport	1.06^***^ (0.43 to 1.69)	0.62^***^ (0.15 to 1.08)	0.59^**^ (0.13 to 1.05)	0.81^***^ (0.36 to 1.25)	1.42^***^ (0.93 to 1.91)
Recreation	0.19^***^ (0.05 to 0.34)	0.01 (−0.13 to 0.14)	0.02 (−0.11 to 0.16)	0.07 (−0.07 to 0.21)	0.05 (−0.10 to 0.20)
Miscellaneous	0.92^***^ (0.43 to 1.41)	0.73^***^ (0.24 to 1.22)	0.35 (−0.15 to 0.86)	0.98^***^ (0.38 to 1.59)	0.95^**^ (0.23 to 1.67)
Other medical	0.26 (−0.14 to 0.67)	0.44^**^ (0.08 to 0.80)	0.29 (−0.06 to 0.65)	0.28 (−0.14 to 0.69)	0.77^***^ (0.21 to 1.34)

Values are presented as percentage points (95% CI). ^***^p<0.01, ^**^p<0.05, ^*^p<0.1. ‘Other medical’ is the total medical expenditures less BPD medication expenses. Quintile 1 refers to households in consumption quintile 1.

**Figure 1:**
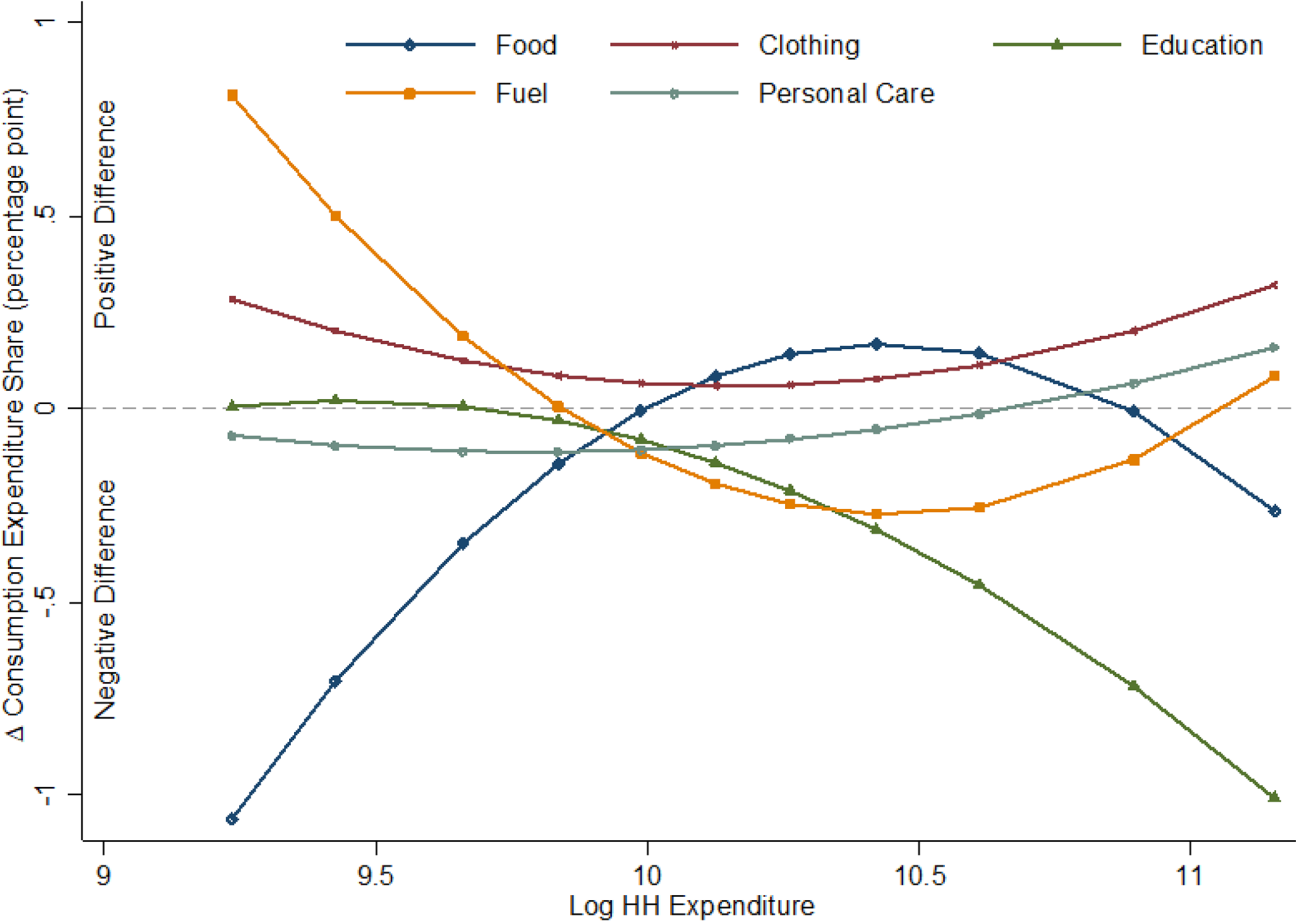
Adjusted differences in consumption share by household expenditure. The adjusted differences are calculated using equation 4 for household expenditures at the 5, 10, 20, 30, 40, 50, 60, 70, 80, 90 and 95^th^ percentiles. Coefficient estimates for α_1_, α_3_ and α_5_ from equation 1 are statistically significant for the consumption categories (i.e. food, clothing, education, fuel, and personal care) illustrated in the figure. Adjusted difference < 0 means households consuming BPD medication on average allocate less resources to the consumption category than households not consuming BPD medication.

Regression results for adjusted differences are reported in Table 3. Estimates for α_1_, α_3_ and α_5_ are individually significant for food, clothing, education, fuel and electricity and personal care and jointly significant for all consumption categories except for recreation. Parameter α_1_ jointly determines, along with parameters α_3_ and α_5_ at certain levels of household expenditures, the adjusted differences in the average consumption share between BPD medication–consuming and not-consuming households. Estimated adjusted differences (for consumption categories for which α_1_, α_3_ and α_5_ are individually significant) are presented in Figure 1. It shows that adjusted differences in the food share are negative for households at the bottom as well as at the top of the household consumption expenditure distribution, and positive for households in the middle. The magnitude of the difference, however, is greater for poorer households. BPD medication–consuming households at the 5th percentile of household expenditures allocate 1.06 percentage points less of their household budget for food compared with households not consuming BPD medication, while at the 95th percentile it is 0.26 percentage points less and at the 50th percentile it is 0.08 percentage points more.

**Table 3:** SUR estimation results for adjusted differences in consumption share

Variable	Food	Tobacco	Clothing	Housing	Education	Fuel	Personal care	Transport	Recreation	Other medical
BPD dummy	−92.255^***^ (−160.562 to −23.948)	−1.942 (−18.158 to 14.273)	27.318^*^ (−1.488 to 56.123)	5.147 (−42.973 to 53.267)	−31.672^*^ (−67.492 to 4.148)	79.541^***^ (48.046 to 111.035)	13.656^*^ (−1.852 to 29.165)	9.769 (−22.177 to 41.715)	2.545 (−7.801 to 12.891)	3.766 (−21.263 to 28.795)
BPD dummy^*^ ln HH exp.	17.706^***^ (4.456 to 30.955)	0.181 (−2.964 to 3.326)	−5.366^*^ (−10.953 to 0.221)	−0.927 (−10.260 to 8.407)	6.708^*^ (−0.240 to 13.655)	−15.269^***^ (−21.378 to −9.160)	−2.816^*^ (−5.824 to 0.192)	−2.080 (−8.277 to 4.116)	−0.526 (−2.533 to 1.481)	−0.727 (−5.582 to 4.128)
BPD dummy^*^ ln HH exp.^2^	−0.848^***^ (−1.490 to −0.206)	0.001 (−0.152 to 0.153)	0.264^*^ (−0.006 to 0.535)	0.038 (−0.414 to 0.490)	−0.355^**^ (−0.691 to −0.018)	0.730^***^ (0.434 to 1.026)	0.144^*^ (−0.002 to 0.290)	0.111 (−0.189 to 0.411)	0.027 (−0.070 to 0.124)	0.040 (−0.195 to 0.275)
Constant	311.223^***^ (269.723 to 352.723)	−13.529^***^ (−23.380 to −3.677)	−66.581^***^ (−84.081 to −49.080)	−125.481^***^ (−154.716 to −96.246)	81.596^***^ (59.834 to 103.359)	−40.341^***^ (−59.476 to −21.207)	−16.552^***^ (−25.974 to −7.130)	−62.537^***^ (−81.946 to −43.128)	−1.955 (−8.240 to 4.331)	−10.244 (−25.450 to 4.963)
Observations	24 235	24 235	24 235	24 235	24 235	24 235	24 235	24 235	24 235	24 235
R^2^	0.390	0.085	0.125	0.137	0.328	0.140	0.130	0.213	0.039	0.059

Values are presented as estimation (95% confidence interval). ^***^p<0.01, ^**^p<0.05, ^*^p<0.1. HH exp. refers to household expenditures, which is the total expenditures less BPD medication spending, and is instrumented by household income. Control variables (not reported in the table) include log household expenditure, log household expenditure squared, share of children <5 y of age, share of elderly (age >65 y), whether household has school-age children (ages 6–15 y), whether household has reproductive age females (ages 15–49 y), household size, household head’s education, whether household has a gas connection, household’s source of drinking water, household’s type of toilet, whether household has computer/laptop/tablet, whether household has internet access, whether household receives a BISP cash transfer, whether household receives remittance from abroad, household’s agricultural production, whether household owns livestock, whether household owns poultry, whether household borrows for consumption and province/region fixed effect.

Adjusted differences in the clothing share are positive at all household expenditure levels. For education, adjusted differences are negative for all except the poor households. At the 95th percentile of household expenditures, the average share of education is 1 percentage point lower for BPD medication–consuming households. Adjusted differences in the personal care consumption share, on the other hand, are negative for households at the bottom and middle of the household expenditure distribution and positive for households at the top. Lastly, adjusted differences in the fuel and electricity share are positive at the bottom and top and negative in the middle.

**Figure 2:**
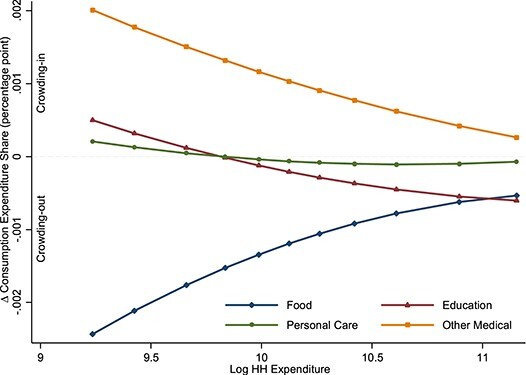
Crowding out of consumption by household expenditure. Values in the vertical axis (y-axis) are calculated using equation 6 for household expenditures at the 5, 10, 20, 30, 40, 50, 60, 70, 80, 90 and 95^th^ percentiles. Coefficient estimates for β_1_, β_3_ and β_5_ from equation 3 are statistically significant for the consumption categories (i.e. food, clothing, education, fuel, and personal care) illustrated in the figure. Values in the y-axis refer to change in consumption expenditure share for the respective category due to change in BPD medication expenditure by Rs1. Negative values in the y-axis refer to crowding out, and conversely positive values refer to crowding in.

Table 4 reports the regression results for the crowding out effect. Estimates of β_1_, β_3_ and β_5_ are individually significant for food, education, personal care and other medical and jointly significant for all consumption categories except for tobacco, housing, fuel and recreation. Similar to the parameters in the adjusted difference equation, parameters β_1_, β_3_ and β_5_ jointly determine the crowding out effects at certain levels of household expenditures. Estimates of crowding out effects are presented in Figure 2. It shows that BPD medication expenditures are associated with crowding out of food consumption and crowding in of other medical consumption at every household expenditure level. However, the magnitude of crowding out and crowding in effects are larger for poorer households. BPD medication expenditures are also associated with crowding out of education and personal care for nonpoor households (i.e. households in the middle or at the top of the expenditure distribution) and crowding in of education and personal care for poor households (i.e. households at the bottom of the expenditure distribution). At the 50th percentile of household expenditures, a Rs100 increase in BPD medication expenditures is associated with a 0.12 percentage point decrease in the food expenditure share, a 0.02 percentage point decrease in the education expenditure share, a 0.01 percentage point decrease in the personal care expenditure share and a 0.10 percentage point increase in the other medical expenditure share.

**Table 4:** SUR estimation results for crowding out effect of BPD expenditure

	Food	Tobacco	Clothing	Housing	Education	Fuel	Personal Care	Transport	Recreation	Medical
BPD exp.	−0.052^*^ (−0.105 to 0.000)	−0.006 (−0.018 to 0.007)	−0.002 (−0.024 to 0.020)	−0.011 (−0.048 to 0.026)	0.028^**^ (0.000 to 0.056)	0.015 (−0.009 to 0.039)	0.017^***^ (0.006 to 0.029)	−0.009 (−0.034 to 0.015)	−0.006 (−0.014 to 0.002)	0.029^***^ (0.010 to 0.048)
BPD exp.^*^ ln HH exp.	0.009^*^ (−0.001 to 0.019)	0.001 (−0.001 to 0.003)	0.001 (−0.003 to 0.005)	0.002 (−0.005 to 0.008)	−0.005^*^ (−0.010 to 0.000)	−0.003 (−0.007 to 0.002)	−0.003^***^ (−0.005 to −0.001)	0.002 (−0.003 to 0.006)	0.001 (−0.000 to 0.002)	−0.005^***^ (−0.008 to −0.001)
BPD exp.^*^ ln HH exp.^2^	−0.000^*^ (−0.001 to 0.000)	−0.000 (−0.000 to 0.000)	−0.000 (−0.000 to 0.000)	−0.000 (−0.000 to 0.000)	0.000^*^ (−0.000 to 0.000)	0.000 (−0.000 to 0.000)	0.000^***^ (0.000 to 0.000)	−0.000 (−0.000 to 0.000)	−0.000 (−0.000 to 0.000)	0.000^**^ (0.000 to 0.000)
Constant	287.674^***^ (251.619 to 323.729)	−11.432^***^ (−20.002 to −2.861)	−56.452^***^ (−71.668 to −41.237)	−128.562^***^ (−154.002 to −103.122)	67.737^***^ (48.802 to 86.672)	−20.609^**^ (−37.258 to −3.961)	−13.287^***^ (−21.481 to −5.093)	−58.385^***^ (−75.262 to −41.508)	−0.705 (−6.174 to 4.763)	−2.336 (−15.533 to 10.861)
Observations	24 235	24 235	24 235	24 235	24 35	24 235	24 235	24 235	24 235	24 235
R^2^	0.392	0.085	0.126	0.137	0.327	0.140	0.131	0.213	0.039	0.064

Values are presented as estimation (95% confidence interval). ^***^p<0.01, ^**^p<0.05, ^*^p<0.1. HH exp. refers to household expenditures, which is the total expenditures less BPD medication spending, and is instrumented by household income. Control variables (not reported in the table) include log household expenditure, log household expenditure squared, share of children <5 y of age, share of elderly (age >65 y), whether household has school-age children (ages 6–15 y), whether household has reproductive age females (ages 15–49 y), household size, household head’s education, whether household has a gas connection, household’s source of drinking water, household’s type of toilet, whether household has computer/laptop/tablet, whether household has internet access, whether household receives a BISP cash transfer, whether household receives remittance from abroad, household’s agricultural production, whether household owns livestock, whether household owns poultry, whether household borrows for consumption and province/region fixed effect.

As a robustness check, we combine equations 3 and 5 and estimate the system of equations with both the BPD medication spending dummy and BPD medication expenditure in the same model. This type of specification was previously used to estimate the crowding out effect of tobacco consumption.^23^ Under this specification, estimates of α_1_, α_3_ and α_5_ are individually significant for food, clothing, education and fuel and jointly significant for all categories except clothing, transport and recreation. On the other hand, the estimates of β_1_, β_3_ and β_5_ are individually significant for education, personal care and other medical and jointly significant for all categories except tobacco, housing, fuel and recreation. The estimates of adjusted differences and crowding out under this combined specification are very similar to those obtained under separate specifications.

## Discussion

Management and treatment of NCDs like hypertension and diabetes, in the absence of adequate health insurance, public provisioning and donor finances, could lead to very high OOP expenditures for households. This has consequences on household resource allocation decisions, which could adversely impact human capital investments necessary for long-term potentials for prosperity. This study shows that BPD medication expenditures constitute a nontrivial share of household expenditures for the BPD medication–consuming households in Pakistan and is associated with allocating relatively smaller budgetary shares for essential expenditure items, such as food and education, compared with BPD medication not-consuming households. We find that BPD medication expenditures are associated with crowding out of food consumption and crowding in of other medical consumption throughout the household expenditure distribution.

However, we do not investigate the causal channels through which hypertension and diabetes could impact household income and resource allocation. Nor do we analyze the behavioral impacts (e.g. food consumption behavior) or comorbidities associated with incidences of hypertension and diabetes. Such analysis would require observing household-level attributes both pre- and postdisease. Given the cross-sectional nature of our data, it is beyond the scope of this article to analyze these issues. For the same reason, we cannot analyze the depletion of the household’s stock of wealth and loss of income associated with these two conditions. These are some of the limitations of this study. In this case, the notion of household resource allocation should be perceived in relation to the flow of income and expenditures on nondurable commodities.

Another limitation of the study is that BPD medication consumption data are self-reported, with a recall period of 1 y. Therefore enumeration errors or misreporting could cause biases in the crowding out estimates. The two most common cases of misreporting could be reporting a higher or lower amount than the actual expenditure and reporting zero expenditure although BPD medication expenses were actually incurred. The first may occur because of not being able to recall the expenditure details, while the latter may result from BPD medication expenses being embedded in physician consultation fees or hospital charges. Therefore our results should be interpreted with caution, keeping in mind these potential reporting issues.

Crowding out of food consumption could have implications for the physiological and cognitive development of children, particularly for poorer households.^25^ Undernutrition is a major cause of infant and child mortality in Pakistan and the country is suffering from a severe problem of nutrition insecurity (i.e. lack of critical vitamins and minerals in diets).^26^ BPD medication expenses could further intensify the nutrition insecurity of BPD-afflicted households by reducing their food expenditures. The crowding in of other medical care consumption, i.e. higher OOP other medical expenditures of BPD-consuming households, may refer to probable comorbidity or multimorbidity incidences in these households. The larger magnitudes of concurrent crowding out of food and crowding in of other medical consumption for households at the bottom of the expenditure distribution suggest aggravating consequences for poor households.

Households in the middle or at the top of the expenditure distribution experience crowding out of education and personal care. The same effect is not evident for poor households, which could be due to a combination of factors, including very high expenditure share of food consumption and very low share of education expenditures. Crowding out of education could have adverse consequences for human development in Pakistan, where expected years of schooling is the lowest in South Asia.^27^ Our study thus demonstrates how hypertension and diabetes might put a strain on limited household budgets and could thwart households’ potential for prosperity. The analysis therefore informs policymakers in evaluating poverty alleviation strategies through low-cost healthcare, preventive health promotions and affordable treatment for hypertension and diabetes.

## Conclusions

Studies examining the impact of OOP treatment costs of noncommunicable diseases on households mainly focus on three aspects: incidences of catastrophic spending, impoverishment and households’ coping strategies. These studies found higher OOP spending for households reporting NCD conditions and a greater likelihood of NCD-afflicted households incurring catastrophic health expenditures and impoverishment. Some studies also reported that NCD-afflicted households often rely on selling assets or borrowing to finance OOP treatment costs. However, no previous studies have assessed the relationship between OOP treatment costs of major NCDs and adjustments in household resource allocation in LMICs. This study is the first to assess the crowding out effects of OOP BPD medication expenses. Our results will help facilitate an understanding of how households modify consumption decisions and reallocate resources across broad commodity groups in response to OOP BPD medication expenses. We detect the commodity groups for which households modify resource allocations in relation to BPD medication expenses. Our findings suggest that BPD medication expenses in Pakistan are associated with allocating relatively smaller household budget shares to essential expenditure items like food and education, which could have detrimental impacts on nutrition and child development. Understanding the association between OOP treatment costs of NCDs and household consumption can thus be helpful in articulating integrated healthcare policies in LMICs.

## Notes

**Authors’ contributions:** All the authors were involved in planning the study and interpretation of the results. BKD and MJH conducted the data analysis and wrote the first draft of the paper. SF contributed to the literature review and discussion.

## Notes

**Funding:** None.

## Notes

**Ethical approval:** Not required.

## References

[1] United Nations . The future we want – outcome document. New York: United Nations Department of Economic and Social Affairs; 2012. http://sustainabledevelopment.un.org/futurewewant.html (accessed 28 August, 2018).

[2] Clark H . NCDs: a challenge to sustainable human development. Lancet. 2013;381(9866):510–1. 23410604 10.1016/S0140-6736(13)60058-6

[3] World Health Organization . Global status report on noncommunicable diseases 2014. Geneva:World Health Organization; 2012. 10.1161/STROKEAHA.115.008097 25873596

[4] Abegunde DO, Mathers CD, Adam T, Ortegon M, Strong K. The burden and costs of chronic diseases in low-income and middle-income countries. Lancet. 2007;370(9603):1929–38. 10.1016/S0140-6736(07)61696-1 18063029

[5] World Health Organization . Non-communicable diseases: key facts. Geneva: World Health Organization; 2018. http://www.who.int/en/news-room/fact-sheets/detail/noncommunicable-diseases (accessed 28 August 2018).

[6] GBD 2017 DALYs and HALE Collaborators . Global, regional, and national disability-adjusted life-years (DALYs) for 333 diseases and injuries and healthy life expectancy (HALE) for 195 countries and territories, 1990–2016: a systematic analysis for the Global Burden of Disease Study 2016. Lancet. 2017;390(10100):1260–344. 28919118 10.1016/S0140-6736(17)32130-X PMC5605707

[7] World Health Organization . A global brief on hypertension: silent killer, global public health crisis: World Health Day 2013. In: WHO/DCO/WHD/2013.2. Geneva:World Health Organization; 2013.

[8] Bloom DE, Cafiero ET, Jané-Llopis E, et al. The global economic burden of noncommunicable diseases. Geneva: World Economic Forum; 2011.

[9] Tolla MT, Norheim OF, Verguet S, et al. Out-of-pocket expenditures for prevention and treatment of cardiovascular disease in general and specialised cardiac hospitals in Addis Ababa, Ethiopia: a cross-sectional cohort study. BMJ Glob Health. 2017;2(2):e000280. 10.1136/bmjgh-2016-000280 PMC5584490 29242752

[10] Alwan A, MacLean DR. A review of non-communicable disease in low-and middle-income countries. Int Health. 2009;1(1):3–9. 24036289 10.1016/j.inhe.2009.02.003

[11] Jan S, Laba TL, Essue BM, et al. Action to address the household economic burden of non-communicable diseases. Lancet. 2018;391(10134):2047–58. 29627161 10.1016/S0140-6736(18)30323-4

[12] Datta BK, Husain MJ, Husain MM, Kostova D. Noncommunicable disease-attributable medical expenditures, household financial stress and impoverishment in Bangladesh. SSM Popul Health. 2018;6:252–8. 30417068 10.1016/j.ssmph.2018.10.001 PMC6214871

[13] Pal R . Out-of-pocket health expenditure: impact on the consumption of Indian households. Oxford Dev Stud. 2013;41(2):258–79.

[14] Kumara AS, Samaratunge R. Impact of ill-health on household consumption in Sri Lanka: evidence from household survey data. Soc Sci Med. 2017;195:68–76. 29154182 10.1016/j.socscimed.2017.11.015

[15] Saleem F, Hassali AA, Shafie AA. Hypertension in Pakistan: time to take some serious action. Br J Gen Pract. 2010;60(575):449–50. 20529498 10.3399/bjgp10X502182 PMC2880743

[16] Wasay M, Jabbar A. Fight against chronic diseases (high blood pressure, stroke, diabetes and cancer) in Pakistan; cost-effective interventions. J Pakistan Med Assoc 2009;59(4):196–7. 19402275

[17] World Health Organization . Diabetes country profiles 2016. Geneva: World Health Organization; 2016. http://www.who.int/diabetes/country-profiles/pak_en.pdf (accessed 2 November 2018).

[18] Pakistan Bureau of Statistics . Pakistan national health accounts 2013–14. Islamabad: Government of Pakistan Statistics Division; 2016.

[19] Datta BK, Husain MJ, Asma S. Assessing the relationship between out-of-pocket spending on blood pressure and diabetes medication and household catastrophic health expenditure: evidence from Pakistan. Int J Equity Health. 2019;18:9. 30646905 10.1186/s12939-018-0906-x PMC6334430

[20] Pakistan Bureau of Statistics. Household integrated economic survey (HIES) 2015–16 . Islamabad: Government of Pakistan Statistics Division; 2017.

[21] Pollak RA . Conditional demand functions and consumption theory. Q J Econ. 1969;83(1):60–78.

[22] Browning M, Meghir C. The effects of male and female labor supply on commodity demands. Econometrica. 1991;59(4):925–51.

[23] John RM . Crowding out effect of tobacco expenditure and its implications on household resource allocation in India. Soc Sci Med. 2008;66(6):1356–67. 18187245 10.1016/j.socscimed.2007.11.020

[24] Banks J, Blundell R, Lewbel A. Quadratic Engel curves and consumer demand. Rev Econ Stat. 1997;79(4):527–39.

[25] Victora CG, Adair L, Fall C, et al. Maternal and child undernutrition: consequences for adult health and human capital. Lancet. 2008;371(9609):340–57. 18206223 10.1016/S0140-6736(07)61692-4 PMC2258311

[26] United Nations Children’s Fund . Situation analysis of children in Pakistan 2017. New York:United Nations Publications; 2017.

[27] United Nations Development Programme . Human development report 2016: human development for everyone. New York: United Nations; 2016.

